# Isolation and Chemical Structural Characterisation of a Compound with Antioxidant Activity from the Roots of *Senna italica*

**DOI:** 10.1155/2013/519174

**Published:** 2013-06-17

**Authors:** Matlou Phineas Mokgotho, Stanley Sechene Gololo, Peter Masoko, Ladislaus Kakore Mdee, Vusi Mbazima, Leshwene Jeremiah Shai, Victor Patrick Bagla, Jacobus Nicolaas Eloff, Leseilane Mampuru

**Affiliations:** ^1^Department of Biochemistry, Microbiology and Biotechnology, University of Limpopo, Turfloop Campus, Private Bag X1106, Sovenga 0727, South Africa; ^2^Department of Pharmacy, University of Limpopo, Turfloop Campus, Private Bag X1106, Sovenga 0727, South Africa; ^3^Department of Biomedical Sciences, Faculty of Science, Tshwane University of Technology, Private Bag X680, Pretoria 0001, South Africa; ^4^Faculty of Veterinary Science, Department of Paraclinical Science, Phytomedicine Programme, University of Pretoria, Onderstepoort, Pretoria 0001, South Africa

## Abstract

*Senna italica*, a member of the Fabaceae family (subfamily Caesalpiniaceae), is widely used in South African traditional medicine to treat a number of disease conditions. Aqueous extracts of the plant are mainly used to treat sexually transmitted infections and intestinal complications. The roots of *S. italica* were ground to a fine powder and sequentially extracted with n-hexane, dichloromethane, acetone, and methanol using serial exhaustive extraction (SEE) method. Thin layer chromatography was used to analyse the phytochemical composition of the extracts and DPPH radical scavenging method to detect the presence of antioxidant compounds. The bioassay guided fractionation of the acetone fraction afforded an antioxidant compound with free radical scavenging activity. The isolated compound was subsequently identified as 3,4′,5-trihydroxystilbene (resveratrol). This study represents the first report of the stilbene resveratrol in *S. italica*.

## 1. Introduction

Plants have formed the basis of sophisticated traditional medicinal systems that have been in existence for thousands of years, and they continue to provide humanity with new remedies [1]. The importance of plants in human and animal health is evident in the increasing presence of natural product drugs in modern medicine. Indeed, natural products and their derivatives represent more than 50% of all the drugs in clinical use in the world today. During the last 40 years, at least a dozen potent drugs have been derived from flowering plants. One example is that of diosgenin, a drug template derived from *Dioscorea* species and used to synthesise contraceptive agents [2]. Other examples include anticancer agents derived from *Catharanthus roseus* as well as laxative agents from *Cassia* species [1]. 

The current advancement in science has made it possible for the isolation of compounds of medical importance from plants that are used in traditional medicinal practices. Different parts of plants such as leaves, roots, stems barks and rhizomes are often extracted using different solvents. In most cases, extracts have been shown to be biologically active both in the *in vitro* and *in vivo* test systems [3]. Some plant-derived compounds are effective in combination with others, while others are active as single entities [4]. However, the isolation of compounds remains a challenging and a mammoth task. Conventionally, the isolation of bioactive compounds is preceded by the determination of the presence of such compounds within plant extracts through a number of bioassays.

*Senna italica*, a member of the Fabaceae family (subfamily Caesalpinaceae), is known for its therapeutic properties in African folk medicine. Some *Senna species* from Venda in South Africa are used to treat sexually transmitted infections (STIs) while others are considered to possess significant antibacterial activities [5]. In the northern parts of the Limpopo province of South Africa, *S. italica *is indicated for the treatment of STIs [6]. A literature survey on the chemical constituents of the genus *Senna* revealed the presence of alkaloids, quinines, and anthraquinones. These types of compounds have been isolated from heartwood, seeds, root bark, roots, and leaves of the genus *Senna* [7]. Other studies have reported the isolation of many other compounds including beta-sitosterol, stigmasterol, and amyrin from the aerial parts of *S. italica* [8]. We have previously reported the antibacterial effect of the acetone extract of *S. italica* on selected bacterial strains, total phenolic content as well as its effect on cancerous Jurkat T cell [9]. Based on these findings, *S. italica* was further investigated with the aim of identifying the compound(s) that exerted the observed biological activities. Antioxidant compounds are important in the prevention or treatment of various human diseases. Undeniably, reports abound that link free radical scavenging potential and other biological benefits of antioxidant compounds [10]. We here report the isolation of resveratrol, a compound with antioxidant activity, from the acetone root extract of *Senna italica* through bioassay-guided fractionation.

## 2. Materials and Method

### 2.1. Extraction

The roots of *Senna italica* were collected from Bolahlakgomo village (Zebediela subregion, Limpopo province, South Africa). A voucher specimen (UNIN11129) has been deposited in the Larry Leach herbarium of the University of Limpopo. The roots were dried at room temperature and subsequently milled to a fine powder using a grinder (ML 90L4, Monitoring and Control Laboratories (Pty) Ltd., RSA). The milled material (700 g) was serial exhaustively extracted using *n*-hexane, dichloromethane (DCM), acetone and methanol (MeOH) as extractants. The extraction process was repeated three times with 2 litres of each solvent. The extracts were filtered through a Whatman no. 3 filter paper and the filtrates concentrated using a rotary evaporator (Büchi Labotec rotavapor model R-205, Germany). The concentrated extracts were then transferred into preweighed beakers, dried under a stream of cold air, and weighed to determine the resultant mass. 

### 2.2. Free Radical Scavenging Activity

The presence of antioxidant constituents in the various extracts was determined using the method of [11]. Thin layer chromatographic plates were prepared and spotted with the different extracts and eluted in solvents systems of varying polarity, namely, ethyl acetate/methanol/water (EMW) (40 : 5.4 : 5) (polar/neutral); chloroform/ethyl acetate/formic acid (CEF) (5 : 4 : 1) (intermediate polarity/acidic); benzene/ethanol/ammonium hydroxide (BEA) (90 : 10 : 1) (nonpolar/basic). For the determination of antioxidant activity, the plates were sprayed with 0.2% 2,2-diphenyl-1-picryl-hydrazyl (DPPH) (Sigma) in methanol as an indicator. A positive reaction is indicated by the appearance of a yellow spot against a purple background.

### 2.3. Fractionation

**Figure 1:**
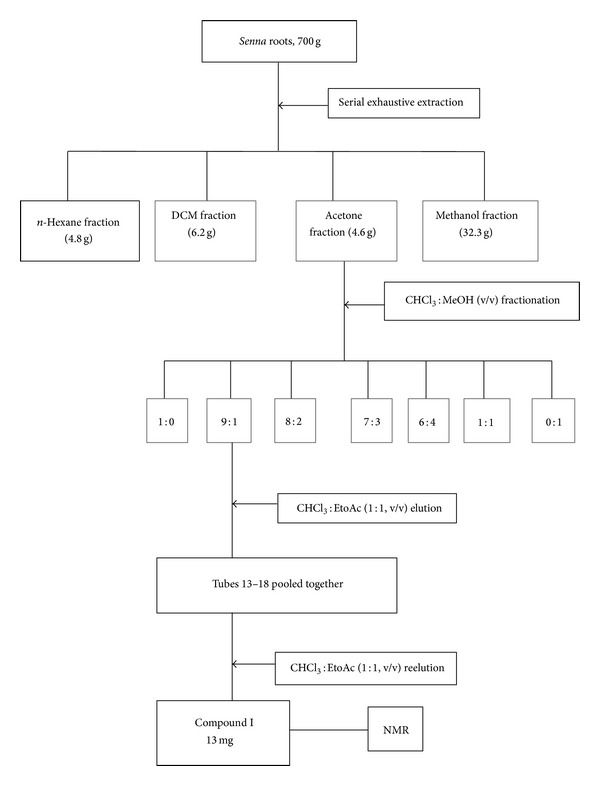
Schematic representation of fractionation of acetone extracts of *S. italica. *

The acetone extract had constituents with antioxidant activity and was thus chosen for further fractionation with CHCl_3_ : MeOH of increasing gradient polarity, starting with 100% CHCl_3_ to 100% MeOH. A 460 mg sample of acetone extract was fractionated (Figure 1) into seven fractions of CHCl_3_ : MeOH in the following ratios: 1 : 0, 9 : 1, 8 : 2, 7 : 3, 1 : 1, 3 : 7, and 0 : 1 (v/v) using column chromatography packed with silica gel 60 (63–200 *µ*m). Following elution, the antioxidant constituents of the resultant fractions were qualitatively evaluated using the TLC-DPPH method described above. The fraction that eluted with 9 : 1 (CHCl_3_ : MeOH (9 : 1)) was found to contain the major compound with antioxidant activity. Hence, the fraction was chosen for further purification of the target compound.

### 2.4. Elution and Structural Characterisation of the Antioxidant Compound

Several mobile phases were tested and fraction F9 : 1 was better resolved with CHCl_3_ : EtAOC (1 : 1). The 9 : 1 (125 mg) was subjected to a silica gel 60 column chromatography using CHCl_3_ : MeOH (4 : 1) as the eluent. Fractions 13–18 were subjected to chromatography using CHCl_3_ : MeOH (9 : 1) as eluent. After recrystallisation, a 13 mg of the pure compound was obtained. The compound was dissolved either in methanol, chloroform, or dimethyl sulfoxide depending on its solubility for analysis. The structure of the isolated compound was elucidated from the data obtained from ^1^H- and ^13^C-NMR spectra.

## 3. Results

Different solvents, depending on their polarity, extract varying quantities of components in crude plant material that may be beneficial or harmful to biological systems. *n*-Hexane, for instance, extracts waxes, fats, and fixed oils while acetone extracts alkaloids, aglycones, and glycosides. On the other hand, methanol extracts sugars, amino acids, and glycosides while DCM will commonly extract alkaloids, aglycones, and volatile oils. For these reasons, plant material was extracted using solvent of varying polarity, namely, hexane, DCM, acetone, and methanol. An increase in the extracted material was observed as the polarity of the extracting solvent increased (Table 1). Methanol extract had the highest percent yield followed by DCM, hexane and acetone yielded the least.

Extracted materials were spotted on TLC plates and eluted in BEA, EMW, and CEF and thereafter sprayed with vanillin-sulphuric acid. The free radical scavenging activity of the extracts was determined using the DPPH assay. The yield of the methanol extract did not correlate with the presence of antioxidant constituents in the extract. Although acetone extracts yielded the least, more active constituents were present in these extracts with strong antioxidant activity. This may possibly be related to the ability of acetone to extract both polar and nonpolar constituents. Since acetone extract demonstrated a superior free radical scavenging activity, it was thus chosen for further purification. The extract was fractionated with the gradient solutions of CHCl_3_ : MeOH of different ratios. Fraction 8 : 2 gave the highest yield (237 mg) followed by fraction 9 : 1 (125 mg), with fraction 0 : 1 giving the lowest yield (14 mg) as shown (Table 2). Fractions 8 : 2 and 9 : 1 contained appreciable amounts of vanillin-reacting compounds than the other fractions, with fraction 9 : 1 showing prominent antioxidant activity than the rest (data not included). Thus, fraction 9 : 1 was selected for further purification.

**Figure 2:**
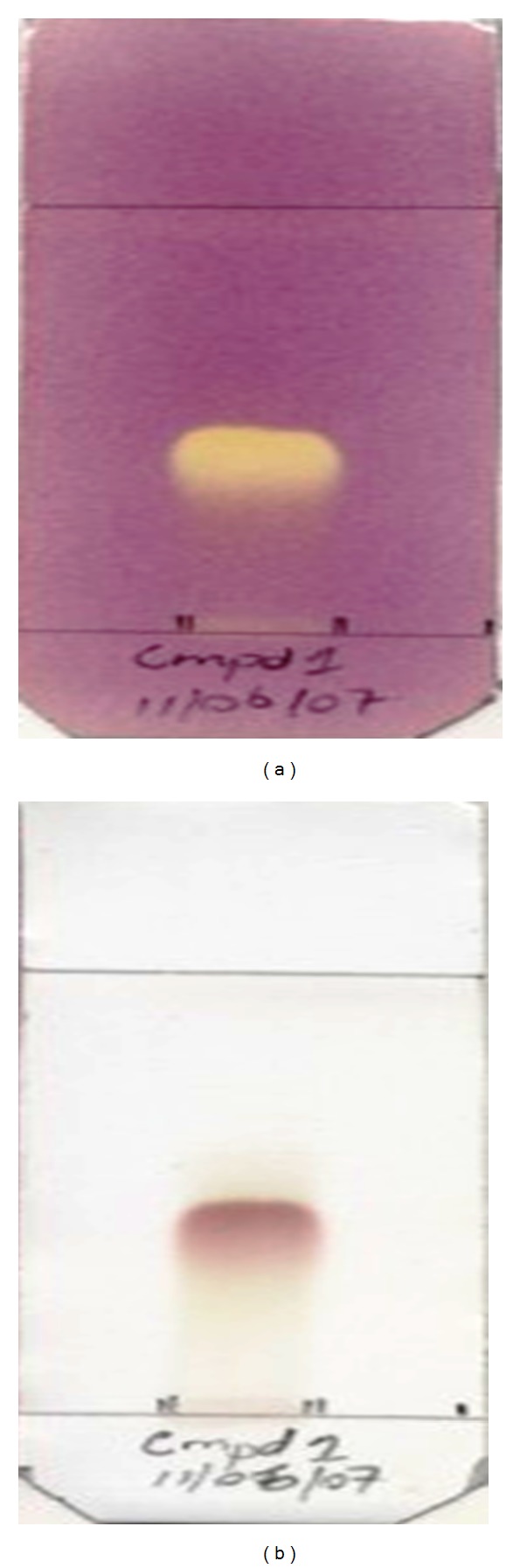
TLC plate of isolated compound sprayed with DPPH (a) and isolated compound sprayed with vanillin-sulphuric acid reagent (b). The plates were developed in CHCl_3_ : EtOAc (1 : 1, v/v) as the mobile phase. A positive reaction is indicated by the appearance of a yellow spot against a purple background.

**Figure 3:**
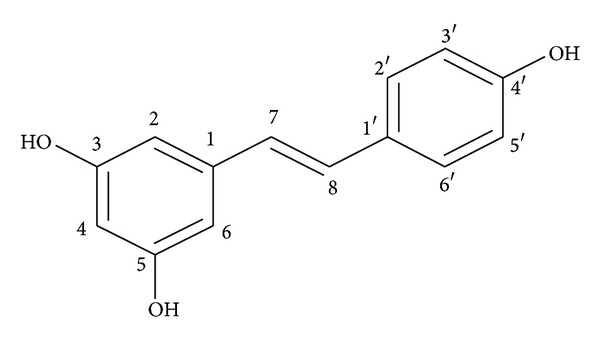
The compound identified as 3,4′,5-trihydroxystilbene (C_14_H_12_O_3_) from ^1^H- and ^13^C-NMR spectra peak assignment.

A suitable mobile phase for elution of the bioactive compound was obtained by comparison of the separated compounds attained from the various ratios of CHCl_3_ : MeOH solution used. The CHCl_3_ : MeOH ratio of 1 : 1 (v/v) gave a better separation of the active compound. The 9 : 1 fraction of the acetone extract was then subsequently eluted with CHCl_3_ : MeOH (1 : 1, v/v) through a silica gel column. Tubes containing similar compounds noted on TLC fingerprint were pooled together and reeluted with CHCl_3_ : MeOH (1 : 1, v/v) through a silica gel column. The reelution of the fraction obtained from the pooled tubes gave yield to a pure compound with a positive free radical scavenging activity evident by a yellow spot against a purple background on TLC following spraying with DPPH (Figure 2). The structure of the isolated compound was elucidated (Figure 3) from the data obtained from ^1^H- and ^13^C-NMR spectra and those obtained from the literature [12, 13] (Table 3). 

Compound 1 was obtained as a white powder that was positive to FeCl_3_ test which indicates the presence of phenolic group. In the 1H-NMR spectrum, the coupling patterns between *δ* 7.43 (2H, d, J = 9.0 Hz) and 6.86 (2H, d, J = 9.0 Hz,) and *δ* 6.55 (2H, brs) and 6.16 (1H, brs) showed the typical resveratrol skeleton [14]. The coupling constant (J = 18.0 Hz) between H-7 (*δ* 6.89, 1H, d) and H-8 (*δ* 7.03, 1H, d) implied that the configuration of compound 1 was in a trans form configuration. In the ^13^C-NMR spectrum, three oxygenated aromatic carbons (*δ* 159.6 (×2) and 158.2) and eleven sp2 carbons (102.7~140.9) were shown. From these data and those presented in Table 3, compound 1 was identified as *trans*-resveratrol [12, 13].

## 4. Discussion and Conclusion

Plant parts such as roots, leaves, stems, and rhizomes possess a myriad of chemical constituents that are biologically active against various disease conditions [3]. In this study, solvents of varying polarity were used in the extraction procedure in an attempt to accommodate the range of polarities of compounds obtained from the roots of *S. italica.*

Methanol extract had the highest yield. The high extract yield obtained with methanol extract could be related to the ability of the solvent to extract compounds of varying polarity [15]. All solvents extracted vanillin-reactive compounds from the roots of *S. italica*. The phytochemical constituents of the extracts were best separated in the intermediate/polar mobile phase, ethylacetate/methanol/water (EMW: 40 : 5.4 : 5), which separates polar and neutral compounds. Acetone extracts showed the presence of antioxidant compounds which were better separated in the intermediate mobile phase (CEF). The free radical scavenging activity demonstrates the hydrogen donating ability on reaction to the stable free radical which results in the decolouration of the DPPH free radical from purple to yellow [16].

A literature survey on the chemical constituents of the genus *Senna *revealed the presence of alkaloids, quinines, and anthraquinones that are mainly polar-based compounds [17]. Resveratrol is a natural polyphenolic compound found in a number of edible plants, especially grapes and peanuts, and has been found to exist in both the *cis* and *trans* isomeric forms. Although the nutritional value and biological activity of resveratrol have been well documented [18], its oligomers are commonly reported to occur in plants belonging to families such as Dipterocarpaceae, Vitaceae, Cyperaceae, Gnetaceae, Welwitschiaceae, Umbelliferae, and Leguminosae [19, 20]. This study represents the first isolation of *trans* resveratrol from the roots of *Senna italica* belonging to the family Fabaceae. The biological effects of resveratrol together with its well-known antioxidant properties are beneficial for the prevention of several diseases. Findings in our previous study [9] showed the presence of substances in the acetone root extract of *S. italica* with antioxidant, antibacterial, and antiproliferative activities while other studies have also confirmed the antiproliferative effect of resveratrol [21]. The presence of resveratrol may possibly explain the observed effect of the acetone crude extracts on cancerous Jurkat T cells in our previous studies [9] and may be the major contributors to biological activity observed in that study.

Structural activity relationships of the antioxidant potential of resveratrol from other studies suggest that the hydroxyl group in 4′ position is not the sole determinant for antioxidant activity. It has also been suggested that the presence of 4′-OH together with its stereoisomer in the *trans-*conformation (4′-hydroxystyryl moiety), is a significant prerequisite in its ability to inhibit cell proliferation [21]. The presence of resveratrol in *S. italica* and the observed antioxidant effect could possibly give more credence to its traditional use in treating various ailments in the Limpopo Province of South Africa [5, 6]. Further studies will focus on the antidiabetic properties of the isolated compound.

## Figures and Tables

**Table 1:** Yields following serial exhaustive extraction of root material of *Senna italica* with *n*-hexane, DCM, acetone, and MeOH.

Extract solvent	Yield (g)	Total yield (g)	Yield (%)
Hexane 1	2.8		
Hexane 2	1.3	4.8	0.69
Hexane 3	0.7		

DCM 1	3.5		
DCM 2	1.5	6.2	0.89
DCM 3	1.2		

Acetone 1	2.3		
Acetone 2	1.4	4.6	0.66
Acetone 3	0.9		

MeOH 1	17.9		
MeOH 2	8.5	32.3	4.6
MeOH 3	5.9		

**Table 2:** Fractionation of the acetone root extract (700 mg) with CHCl_3_ : MeOH different ratios on silica gel chromatography.

Systems	CHCl_3_ (%)	MeOH (%)	Solvent quantity (mL)	Yield (mg)
A	100	—	500	58
B	90	10	700	125
C	80	20	1200	237
D	70	30	500	30
E	50	50	600	25
F	30	70	500	16
G	—	100	600	14

**Table 3:** ^
13^C NMR data of isolated compound (300 MHz, acetone-d_6_).

C number	Literature data	Isolated compound data
1	141.3	140.9
2	105.8	105.7
3	159.7	159.6
4	102.7	102.7
5	159.7	159.6
6	105.8	105.7
7	129.4	129.1
8	127.0	126.9
1′	130.4	130.0
2′	128.8	128.7
3′	116.5	116.4
4′	158.4	158.2
5′	116.5	116.4
6′	128.8	128.7
